# Colorectal anastomotic leak: delay in reintervention after false-negative computed tomography scan is a reason for concern

**DOI:** 10.1007/s10151-017-1689-6

**Published:** 2017-09-19

**Authors:** C. C. M. Marres, A. W. H. van de Ven, L. G. J. Leijssen, P. C. M. Verbeek, W. A. Bemelman, C. J. Buskens

**Affiliations:** 1 Department of Surgery, Flevo Hospital, Hospitaalweg 1, 1315 RA Almere, The Netherlands; 2 Department of Surgery, Academical Medical Center, Amsterdam, The Netherlands

**Keywords:** Colorectal surgery, Computed tomography, Anastomotic leakage, Oncology

## Abstract

**Background:**

Early detection of anastomotic leakage (AL) after colorectal surgery followed by timely reintervention is of crucial importance. The aim of this study was to investigate the accuracy of computed tomography (CT) imaging for AL and the effects of delay in reintervention after a false-negative CT.

**Methods:**

All files from patients who had colorectal surgery with primary anastomoses between 2009 and 2014 were reviewed. The predictive value of CT scanning for AL was determined and correlated with short-term postoperative patient outcomes. In addition, factors predictive of false-negative scans were assessed.

**Results:**

Six hundred and twenty-eight patient files were reviewed. In total, a CT scan was performed in 127 patients. Overall, leakage was seen in 49 patients (7.8%). The positive and negative predictive values were 78 and 88%, respectively. Sensitivity was 73% and specificity 91%. In patients with a true-positive CT (*n* = 24), reintervention followed after a median interval of 0 days (IQR 1), whereas this was 1 day (IQR 2) in the false-negative group (*n* = 11) (*p* < 0.05). This was associated with a significantly increased mortality rate (1/24 = 4.2% vs 5/11 = 45.5%) (*p* < 0.005), an increased length of hospital stay [median 28 days (IQR 26) vs 54 days (IQR 20) (*p* < 0.05)].

**Conclusions:**

Delayed reintervention after false-negative CT scanning is associated with a high mortality rate and a significant increase in length of hospital stay.

## Introduction

Anastomotic leakage (AL) is a life-threatening complication after colorectal surgery [1]. In the literature, mortality rates after AL vary from 15 to 33%. Early detection of AL followed by timely reintervention decreases mortality rates. In daily clinical practice, abdominal computed tomography (CT) scanning is most frequently used to diagnose or exclude AL after colorectal surgery [2–4]. Compared to water-soluble contrast enema and plain X-ray, CT scan is more sensitive and is able to detect other complications such as bleeding, perforation or abscess. At the same time, it can be used to guide therapeutic percutaneous drainage of abscesses.

Considering how commonly CT is performed in postoperative colorectal patients, the literature on the accuracy of CT scanning in patients with AL is scarce. The only systematic review, performed by Kornmann et al. [5], included a total of only 221 abdominal CT scans from eight different studies. This review showed a relatively low sensitivity of 68%, with a large range reported from these small, retrospective studies. The technical quality of the CT scan was often suboptimal (10-mm sections, no enteral or intravenous contrast), and the definition of AL was inconsistent. Previous studies suggested that false-negative CT outcome may delay reintervention [6, 7], but the clinical consequences of a false-negative CT scan were only described in one study [8].

Recent studies by Huiberts et al. [9] and Kauv et al. [10] showed that leakage of contrast medium was the only independent predictive factor for AL. In our institution, abdominal CT scanning with rectal contrast enema (RCE) in cases where there is suspicion of AL has been the standard imaging procedure for over 6 years.

The aim of this study was to investigate the accuracy of abdominal CT scanning with RCE for anastomotic leakage and the effect of false-negative scans on delay in therapeutic intervention and clinical outcome.

## Materials and methods

Data from a prospectively maintained database of all patients who had elective or emergency colorectal surgery with primary anastomoses for malignant or benign disease between 2009 and 2014 were reviewed. Patient characteristics, including age, gender and American Society of Anesthesiologists (ASA) classification, were collated. Type of operation, complications, length of hospital stay, length of stay in the intensive care unit (ICU), indications for abdominal CT scans, timing and outcome of CT scans and subsequent reinterventions were also evaluated.

All colorectal resections were performed by, or under supervision of, a specialist colorectal surgeon. Perioperatively, patients were managed according to standard fast-track protocol [11]. Abdominal CT was performed when patients had signs of sepsis with clinical symptoms and/or physiological deterioration (e.g. deviations in respiratory rate, pulse rate, blood pressure, temperature, urine production or neurological status).

CT imaging was performed on a 16- and 64-sliced-MDCT scanner (Philips, Netherlands), with a slice thickness of algorithm of 3–5 mm with axial and coronal reconstructions. Scanning protocol included intravenous and rectal contrast. CT scans were reviewed by experienced radiologists. For this study, radiology reports were used as given at the time rather than later amendments, in order to evaluate the effect of delay in reintervention after false-negative scans. Our radiologists scored the following features to asses the CT scans: fluid intraabdominally, fluid near the anastomosis, free air in the abdomen, air near the anastomosis and contrast from the lumen. AL was graded according to the definition of the International Study Group of Rectal Cancer [12]. Grade C was defined as a leak requiring surgical reintervention, grade B as a leak requiring percutaneous reintervention, and grade A as a leak requiring antibiotics at the most. Since the indication for antibiotics was not always based on CT scan findings and it had minimal to no clinical impact on the patients postoperative course, only grade B and C anastomotic leaks were included in the CT accuracy analyses.

### Statistical analysis

Statistical analysis was performed using SPSS software, version 22.0 (SPSS Inc, Chicago, IL, USA). Sensitivity, specificity, positive predictive value (PPV) and negative predictive value (NPV) were calculated with 95% confidence intervals (CI). Categorical data are presented as frequencies and percentages compared by the Chi-square test. The parametric and nonparametric continuous data are presented as means and standard deviations and were analysed by the Mann–Whitney *U* test. Missing data for every variable were less than 10%, and therefore there no imputation of missing data was performed. A two-tailed *p* value of <0.05 was considered statistically significant.

## Results

Between 2009 and 2014, 628 patients underwent colorectal surgery with primary anastomosis in our institution. In 127 out of 628 patients, an abdominal CT scan was performed based on clinical symptoms and/or signs of sepsis. Of these patients, 69 (54.3%) were men and 58 (45.7%) women with a median age of 66 years (range 44–89 years).

Ninety-nine of the 127 patients undergoing CT scan for suspicion of AL were given enteral contrast according to protocol (78%). In 85 patients (86%), the contrast reached the anastomosis. Relevant baseline characteristics are summarised in Table 1.

**Table 1:** Demographic characteristics

Variables	All patients *N* (%)^a^	Patients with CT *N* (%)^a^
Age, years		
Median	66	66
Range	18–96	44–89
Sex		
Female	295 (47.0)	58 (45.7)
Male	333 (53.0)	69 (54.3)
ASA class		
ASA 1 or 2	489 (77.8)	92 (72.4)
ASA 3 or 4	132 (21.0)	26 (20.5)
Type of operation		
Right colectomy	203 (32.3)	39 (30.7)
Left colectomy	41 (6.5)	11 (8.7)
Sigmoidectomy/LAR	310 (49.4)	53 (41.7)
Colectomy	37 (5.9)	13 (10.2)
Other	36 (5.8)	11 (8.7)
Open/laparoscopic		
Open	84 (13.4)	40 (31.5)
Laparoscopic	512 (81.5)	87 (68.5)
Stoma		
No stoma	413 (65.8)	88 (69.3)
Loop ileostomy	126 (20.1)	37 (29.1)
End colostomy	5 (0.8)	2 (1.6)
Urgency		
Elective	508 (80.9)	93 (71.9)
Emergency	87 (13.9)	29 (22.70
Anastomotic leak		
Grade C	38 (6.1)	29 (22.8)
Grade B	6 (0.8)	6 (4.7)
Grade A	5 (1.0)	5 (3.9)
Total	628 (100)	127 (100)

*CT* computed tomography, *ASA* American Society of Anesthesiologists, *LAR* low anterior resection

^a^ Unless stated otherwise in the first column

### Anastomotic leakage

Overall leakage after surgery (grade A, B and C) was 7.8% (49 out of 628 anastomoses). Thirty-eight patients had grade C AL (6.0%), six patients (1.0%) had grade B leakage and five patients had grade A leakage (0.8%). Leak rates were comparable for patients with open or laparoscopic procedures and for patients with benign or malignant disease.

In nine patients with AL, no CT was performed prior to reintervention. Six of these patients were reoperated on within 5 days of primary resection without diagnostic imaging because of their clinical condition. In the other three patients, a water-soluble contrast enema with X-ray was performed, during the implementation phase of the abdominal CT scan with contrast enema in 2009.

### Predictive value of the CT scan

Twenty-four patients had a true-positive CT scan, with AL demonstrated at reintervention. Eleven patients had a false-negative scan; there were no signs of AL on CT scan, but a leak was confirmed afterwards during reintervention or autopsy. Seventy-nine patients had a true-negative scan, and eight patients had a false-positive scan. CT scans were counted as false-positive when the scan was reported as showing signs suggestive of AL; however, either during relaparoscopy there was no leakage detected or they had no clinical signs of AL and recovered without any treatment (no antibiotics). In six patients, CT scan revealed too much free air or fluid in the abdomen (based on how much free air or fluid was expected on the corresponding day postoperatively). In one of these patients, relaparoscopy did not show any abnormality. In two patients, minimal contrast was seen near the anastomosis, but the clinical sign was absent and they recovered without any treatment.

The accuracy of CT scanning for AL in this study was 85.0%. The positive and negative predictive values were, respectively, 0.78 (CI 0.65–0.92) and 0.88 (CI 0.82–0.95). The sensitivity was 0.72 (CI 0.59–0.86) and specificity 0.91 (CI 0.85–0.97) (Table 2). The area under the receiver operating characteristic (ROC) curve was 0.80. This was calculated in accordance with the recommendations of Castor et al. [13] for binary diagnostic tests.

**Table 2:** Sensitivity, specificity, positive and negative predictive value for anastomotic leakage

CT outcome	Anastomotic leakage	No anastomotic leakage	Results (95% CI)
Positive	24	8	Sensitivity 0.69 (0.51–0.83) Specificity 0.91 (0.83–0.96)
Negative	11	79	PPV 0.75 (0.56–0.87) NPV 0.88 (0.79–0.94)

Patients with grade A anastomotic leak were excluded

Values are given as number of patients (n). Sensitivity, specificity, PPV and NPV are given with 95% CI

*CT* computed tomography, *PPV* positive predictive value, *NPV* negative predictive value

### Impact on clinical outcome

The overall mortality rate after grade B and C leakage was 20.5% (9 out of 44 patients). Out of 24 patients with a true-positive CT scan, only one died (4.2%), whereas mortality after a false-negative CT was 45.5% (5 out of 11 patients) (*p* < 0.005). In patients with leakage predicted by CT, reintervention was performed after a median interval of 0 days (IQR 1), whereas this was 1 day (IQR 2) in the false-negative group (*p* < 0.05). This resulted in a significantly increased length of hospital stay [median of 28 days (IQR 26) vs 54 days (IQR 20) (*p* < 0.05)]. The median length of stay in the ICU was 3 days (IQR 10) for patients with a true-positive CT scan versus 2 days (IQR 14) in patients with a false-negative scan (Table 3).

**Table 3:** Clinical outcome of anastomotic leakage in patients with grade B and C leakage (*n* = 35)

	Overall *N* = 35	True-positive CT *N* = 24	False-negative CT *N* = 11	*p* value
Mortality (%)	6 (17.1%)	1 (4.2%)	5 (45.5%)	.003*
Length of hospital stay, median (IQR)	30.5 (31)	28 (26)	54 (20)	.014**
Days in ICU, median (IQR)	3 (10)	3 (10)	2 (14)	.094**
Days from operation to CT, median (IQR)	7 (5)	7 (4)	4 (4)	.121**
Days from CT to reintervention, median (IQR)	0 (1)	0 (1)	1 (2)	.011**

*CT* computed tomography scan, *ICU* intensive care unit, *IQR* interquartile range

* Chi-square test

** Mann–Whitney *U* test

To identify predictive factors influencing CT accuracy, the following variables were analysed: anatomical site of the anastomosis, emergency surgery, whether enteral contrast was given and if the contrast had reached the anastomosis on CT scan.

There were no statistically significant differences found in these variables between patients with a true-positive and a false-negative scan. It is noteworthy, however, that the contrast reached the level of the anastomosis in only 66.6% in the false-negative group, but in 85.0% in the true-positive group (Table 4).

**Table 4:** Parameters predicting accuracy of CT scanning for anastomotic leakage

	Overall *N* = 35	True-positive CT *N* = 24	False-negative CT *N* = 11	*p* value
Emergency surgery	11 (31.4%)	7 (41.2%)	4 (36.4%)	.670*
Rectal anastomosis	12 (34.2%)	9 (37.5%)	3 (27.2%)	.554*
Rectal contrast	29 (82.9%)	20 (83.2%)	9 (81.8%)	.466*
Contrast reached anastomosis	23 (79.3%)	17 (85.0%)	6 (66.6%)	.874*

*CT* computed tomography

* Chi-square test

## Discussion

This study demonstrated an 84% accuracy of CT scan for AL after colorectal surgery. A leak was missed in 11/39 patients resulting in a mediocre sensitivity of 72%. False-negative CT was associated with a significantly higher mortality.

The sensitivity and specificity of CT scanning found in this study are in line with the results of the systematic review of Kornmann et al. [5] and with three studies on sensitivity of CT scanning [8–10].

Previous studies also showed that false-negative CT scan causes delay in reintervention after AL, but numbers of patients are small in these reports. Kornmann et al. [8] reported that mortality increased to 63% after delayed diagnosis compared to 7% in patients with a true-positive CT outcome and immediate intervention, which is comparable to our results (45.5 vs 4.2%).

The two most recent studies found that leakage of contrast medium was the only independent predictive factor for AL [9, 10]. Kauv et al. [10] reported that 58 patients were scanned with RCE and 95 without. Of the 11 false-negative or indeterminate CT scans in their study only 2 (18%) were performed with RCE. Huiberts et al. [9] reported that RCE was given to 52 patients and 52 patients received oral contrast only. The contrast reached the anastomosis in only 31% of the patients. In the false-negative group, contrast was present at the site of the anastomosis in 39% of the cases. In our study, 99 patients were given rectal contrast. In 85 patients, the contrast reached the anastomosis (86%). The contrast did not reach the anastomosis in 1/3 of the patients with a false-negative scan.

Although several studies mention that performing a scan too early in the course of AL, i.e. before AL is radiologically detectable, leads to false-negative scans, [6, 7, 14], in the present study no significant difference was found in the timing of the CT scans of patients with a true-positive and patients with false-negative scan.

A limitation of this study is the fact that the indications for CT scanning, timing of the intervention or “wait and see” policy are subject to a surgeon’s personal opinion or experience. The only published prospective study on this subject to date suggested that abdominal complications cannot be predicted by a CT scan on day 5 after laparoscopic colorectal resection and therefore it cannot be recommended for routine use [15].

In previous studies, different definitions of clinical and radiological AL are used. Surprisingly, there is little consensus on the definition of AL, different criteria making interpretation of data more difficult [16, 17]. The definition and grading of AL by the International Study Group of Rectal Cancer [18] were used in this study and should perhaps be used as a standard in future reports.

The strength of this study is the consecutive series from a single, nonacademic, secondary care centre, reflecting daily clinical practice of most colorectal surgeons. To our knowledge, this is the first study with detailed information on clinical outcome after false-negative CT scans. Furthermore, this is one of the largest studies on accuracy of CT scanning for AL with the largest population of patients screened with RCE.

## Conclusions

Our results indicate that a false-negative CT scan in suspected AL is associated with a higher mortality rate and a significant prolonged length of hospital stay. CT scan is not accurate enough to provide assurance of anastomotic integrity and should be considered in the round with other patient parameters with a low threshold for intervention maintained if a negative scan does not fit with symptoms, signs and other results. Diagnostic laparoscopy despite a negative scan should still be considered where there remains clinical suspicion of AL.
